# The Impact of Polyvinylpyrrolidone on Properties of Cadmium Oxide Semiconductor Nanoparticles Manufactured by Heat Treatment Technique

**DOI:** 10.3390/polym8040113

**Published:** 2016-04-08

**Authors:** Naif Mohammed Al-Hada, Elias Saion, Zainal Abidin Talib, Abdul Halim Shaari

**Affiliations:** Department of Physics, Faculty of Science, Universiti Putra Malaysia, 43400 Serdang, Selangor, Malaysia; emansaion@gmail.com (E.S.); zainalat@upm.edu.my (Z.A.T.); ahalim@upm.edu.my (A.H.S.)

**Keywords:** cadmium oxide semiconductor nanoparticles, polyvinyl pyrrolidone, thermal treatment route

## Abstract

Cadmium oxide semiconductor nanoparticles were produced using a water based mixture, incorporating cadmium nitrates, polyvinyl pyrrolidone (PVP), and calcination temperature. An X-ray diffraction (XRD) evaluation was conducted to determine the degree of crystallization of the semiconductor nanoparticles. In addition, scanning electron microscopy (SEM) was conducted to identify the morphological features of the nanoparticles. The typical particle sizes and particle dispersal were analyzed via the use of transmission electron microscopy (TEM). The findings provided further support for the XRD outcomes. To determine the composition phase, Fourier transform infrared spectroscopy (FT-IR) was conducted, as it indicated the existence of not only metal oxide ionic band in the selection of samples, but also the efficient removal of organic compounds following calcinations. The optical characteristics were demonstrated, so as to analyze the energy band gap via the use of a UV–Vis spectrophotometer. A reduced particle size resulted in diminution of the intensity of photoluminescence, was demonstrated by PL spectra. Plus, the magnetic characteristics were examined using an electron spin resonance (ESR) spectroscopy, which affirmed the existence of unpaired electrons.

## 1. Introduction

The growth of organic-inorganic nanomaterials, commonly pursued via the joining of artificial polymers and inorganic particles or by combining altered nanoparticles (NPs) to polymer matrices, is designed to produce materials, which demonstrate enhanced characteristics. The nanomaterials, which consist of both inorganic nanoparticles and organic polymers, can be considered a brand new classification of materials. These new materials demonstrate enhanced features, particularly when placed alongside their microparticle equivalents [1]. Thus, nanomaterials, which incorporate metal oxide nanoparticles, represent great scientific and technological values as a result of the exceptional physical and chemical features deriving from their nanoscale size and increased amount of surface atoms. Due to the fact that their characteristics are reliant on the increased surface area to volume ratio and the quantum confinement effect, their prospective uses span across nearly every discipline and field of human enterprise [2,3,4,5,6,7,8,9,10,11,12]. A polymer matrix is employed as the setting for nanoparticle development, as it synergistically brings together the characteristics of the host polymer matrix and the distinct nanoparticles, which eventually produce within [13,14,15,16,17].

In fact, nanoparticles featuring metal oxides fixed in polymer matrices have drawn a great deal of emphasis and focus, due to the exceptional characteristics demonstrated by the materials. As a result of the nanometer dimensions of the particles, their physicochemical features are very different from the features observed in molecular and bulk materials [18,19,20]. This kind of compound materials is believed to exhibit interesting optical, electrical, magnetic, catalytic, and mechanical characteristics [20,21,22]. A broad selection of techniques employed for the production of metal and metal oxide-polymer nanocomposites and can be found elsewhere [23,24,25,26,27,28,29,30,31,32]. As just one example of these metal oxide nanomaterials, cadmium oxide is a II–VI composite semiconductor made up of cadmium (group II) and oxygen (group VI) from the periodic table of naturally occurring elements [33]. The II–VI semiconductor CdO has a face center cubic (fcc) ionic crystal structure and is thought to be a material with many uses, as it demonstrates a range of intriguing chemical and physical characteristics. CdO is a n-type semiconductor transition metal oxide, which includes narrow direct band gaps of 2.2–2.5 eV [34]. This unique structure results in a range of intriguing characteristics, which mean that CdO nanomaterials can be successfully employed in a range of physical applications [35,36]. They are particularly useful in optoelectronic devices like solar cells, due to their elevated level of pellucidity within the visible region of the solar spectrum [37]. This kind of material is also commonly employed in diodes, gas sensors, and clear electrodes [38,39,40]. As a result of their intriguing characteristics and diverse uses, a range of CdO nanomaterials (for example, nano-Cubes [41], nano-clusters thin films [42], nanowires [43,44], nanoclusters [45], nanoparticles [46,47], nanocrystals [48,49], nanorodes [50], CdO rhombus-like nanostructure [51], Heterojunction Nanofiber [52], and many others [53]) have been produced via the use of different methods. There are a number of methods whereby CdO nanomaterials can be produced. They include, but are not limited to, the sonochemical technique [54], the solvothermal method [55], the sonochemical process [56], heat based decomposition [57], the soft chemical process [58], the evaporation technique [59], and more. Yet, the efficiency of each technique for distributing nanoparticles is restricted by re-accumulation of the separate nanoparticles and the development of an equipoise environment within certain situations, which dictates the dimension levels of the agglomerate of distributed nanoparticles. There are additional restrictions pertaining to temperature and the low level of tolerance of some forms of inorganic nanoparticle to motorized pressures. Those particles, which include a polymer chain, are significantly more reliable in the face of accretion, due to a vast drop in their surface energy; particularly in contrast with plain particles. A polymer can be used as a chain by, initially, producing the inorganic nanoparticles (via any one of the aforementioned methods) and then distributing them within a polymer liquid.

The existence of a polymer chain boosts the synergy of the particles within the polymer matrix, so that it takes less energy to distribute them. The function of polymers as the most valuable substance when it comes to controlling the distribution of nanoparticles, in the face of accretion and as dissolving agents, offers a useful resource for additional regulation over the distributions of various employments.

This study is concerned with the investigation of PVP concentration that regulates production of morphologically distinct CdO nanoparticles. It particularly emphasizes on the diverse structural form of nanoparticles gained via the use of different calcination temperatures and PVP concentrations in manufacturing; including the design of those nanoparticles, which can exhibit multidimensional functions. For example, polymer demonstrates its role as a capping agent and this can play an important part in the formation of metal oxide nanoparticles. Yet, it is also important to note that polymer regulates the growth of nanoparticles via its concentration, reduces the rate of accretion, enhances the crystallinity, and promotes consistency and standardization within the definition of nanoparticles.

A comprehensive method for producing pure CdO nanoparticles discussed here involves a liquid combining nitrate metallic ions and a PVP capping agent. This solution undergoes calcination at a suitable temperature and then it is subject to an examination of its morphological, structural, optical, and magnetic features. If this technique is contrasted with other alternative processes, we see that it is more basic and the raw materials are more affordable. Thus, it has the potential to be employed in additional (more large scale) operations across the field [17,60].

## 2. Experimental Work

### 2.1. Materials

The chemicals employed within the production of the CdO semiconductor nanoparticles were a cadmium nitrate reagent, polyvinylpyrrolidone (PVP), and deionized water. PVP (*M*_W_) = 29,000 g/mol) was employed as a capping agent. It was obtained from Sigma Aldrich (Darmstadt, Germany). Also, cadmium nitrate reagent (*M*_W_ = 308.46 g/mol) was utilized as a metal precursor provided by Acros Organics (New Jersey, NJ, USA). The chemicals involved were all employed as provided and underwent no additional purification.

### 2.2. Synthesis of the Nanoparticles

The various concentrations of PVP (0.03, 0.04 and 0.05 g/mL) were dissolved in 100 mL of deionized water, as a way to prepare the polymer solution before incorporating 0.2 mmol of the cadmium nitrate, Cd(NO_3_)_2_·4H_2_O. This composite solution was consistently stirred for 2 h to reach a state of homogeneity. To entirely eradicate the water, it was moved into a glass receptacle and exposed to 80 °C in an oven for a day. The hard substance, which came out of the oven, was broken up into a powder, after 15 min of work with a mortar and pestle. The mixture then underwent calcination at a temperature of 600 °C for 3 h to eliminate the organic components and attempt to crystallize the CdO nanoparticles [17].

### 2.3. Characterization

A range of characterization methods was employed to examine the features of the produced CdO nanoparticles. The structure was analyzed with the use of an X-ray diffraction (XRD) spectrometer (Shimadzu model 6000, Lelyweg1, Almelo, The Netherlands,). It was necessary to consider Cu *K*α (0.154 nm) as a radiation source, in order to produce diffraction patterns within the crystalline samples at an ambient temperature (in the 2θ range between 10° and 80°). The morphology and the consistency of the samples were investigated via the use of scanning electron microscopy (SEM). The morphology (shape), particle dimensions, particle dimension distribution, and the consistency of the nanoparticles were identified via the use of transmission electron microscopy (JEOL TEM model 2010F UHR, Munich, Germany), operated at an accelerating voltage of 200 kV. The infrared spectra (280–4000 cm^−1^) values were observed using a Fourier transform infrared (FTIR) spectrometer (Perkin Elmer model 1650, Labexchange, Swabian Burladingen, Germany), in order to reaffirm the eradication of the capping agent and to examine the inorganic composite of CdO remaining after calcination. In addition, a UV-vis spectrophotometer (Shimadzu model UV-3600, Kyoto, Japan) and photoluminescence (PL) (Perkin Elmer LS 55, Waltham, MA, USA) were employed to analyze the optical characteristics of the samples at ambient temperatures, within the range of 200–800 nm. The magnetic characteristics were examined via the use of electron spin resonance (ESR) spectroscopy (JEOL-JES-FA200, JEOL, Japan) at an ambient temperature.

## 3. Results and Discussion

### 3.1. Effect of Calcinations Temperature on Structural, Morphology, Phase Composition, Optical Properties

This part of the report succinctly outlines the findings of an earlier study [17] which the authors conducted on the impact of calcinations temperature on structure, morphology, phase composition, and the optical characteristics of CdO nanoparticles. If the precursor of CdO nanoparticles calcined at 500, 550, 600, and 650 °C, the particle sizes increased to 23, 28, 37, and 39 nm respectively, as demonstrated by TEM and XRD evaluations. The point of total crystallization was observed at 500 and beyond. This was affirmed by the complete lack of organic absorption band of PVP and retaining inorganic absorption band of CdO within the FT-IR spectrum. The optical investigations demonstrated that the energy band gap of the CdO nanoparticles decreased with an increase in temperature. The prime calcinations temperature of CdO nanoparticles was observed at 600 °C, as this temperature turned out to be the lowest temperature at which the nanoparticles could still be pure. Also, at this temperature, CdO semiconductor nanoparticles exhibited the smallest particle sizes and an almost entirely consistent dispersion of form.

### 3.2. Impact of PVP Concentration on Structure, Morphology, Phase Composition, Optical, and Magnetic Properties

#### 3.2.1. Structural Characterization

The XRD peaks of CdO nanoparticles, which were prepared at a range of concentrations of PVP (from 0 to 0.05 g/mL) and calcined at 600 °C temperatures, were examined to identify the function of PVP within the production of CdO nanoparticles.

**Figure 1:**
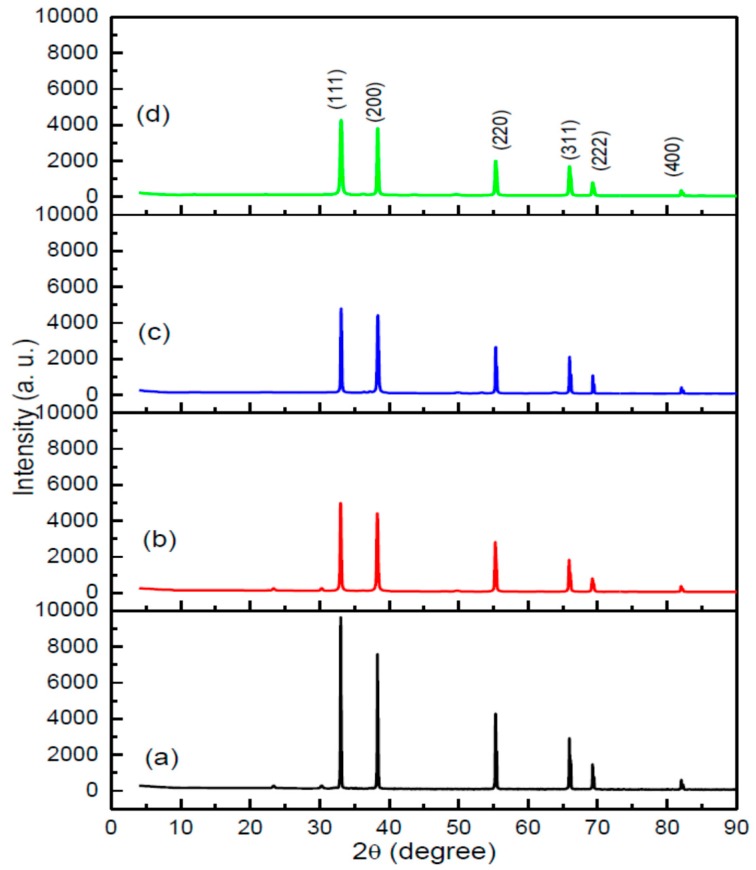
XRD patterns of as-prepared and calcined CdO nanoparticles at PVP different concentration of (**a**) 0.00; (**b**) 0.03; (**c**) 0.04 and (**d**) 0.05 g/mL.

Figure 1 demonstrates typical XRD patterns, both in combination with and without PVP. Figure 1a shows a sample without PVP, which calcined at 600 °C. The spectrum displays sharper, narrower diffraction peaks and a mixed phase of CdO and CdCO_3_ at 24° and 30°, indicating that a crystalline CdO structure has been formed. The presence of numerous diffraction peaks—of (111), (200), (220), (311), (222), and (400)—within the diffraction patterns indicates that the CdO samples have a typical face centered cubic (FCC) structure, referring to the PDF Card No: 005-0640 data [17].

Figure 1b–d represents typical XRD patterns of CdO nanoparticles when combined with 0.03 g/mL of PVP at 600 °C calcination. The spectrum displays sharper and narrower diffraction peaks, indicating that a crystalline CdO nanoparticles structure has been formed. In addition, elevated values of PVP were at recorded at the smallest crystallinity of the CdO nanoparticles by decreasing the intensity of peaks and eradicating the unwanted smaller peaks, which emerged without PVP. This crystallinity improvement, alongside rising amount of PVP, is restricted by the reduction of the crystalline, as observed in TEM results. In Table 1, it can also be seen that the crystalline size has lowered, alongside rising amount of PVP. The presence of numerous diffraction peaks of (111), (200), (220), (311), (222), and (400) within the diffraction patterns indicates that the CdO samples have a typical face centered cubic (FCC) structure, referring to the PDF Card No: 005-0640 data.

The most extreme peak of CdO nanoparticles was allocated to the (111) index plane at 2θ = 33.1°. The average crystalline size of the CdO nanoparticles was identified according to the expansion of the most extreme peak (111), via the use of the highly regarded Debye-Scherrer Equation (1). The equation is presented below:

(1) *D* = 0.9λ/βcosθ

where *D* is the crystallite size (nm), β is the full width of the diffraction line at half of the maximum intensity *i.e.*, (111), which is calculated in radians. λ is the X-ray wavelength of Cu *K*α = 0.154 nm and θ is the Bragg’s angle [61]. The crystalline sizes of CdO nanoparticles were observed to span from 38 to 22 nm, alongside a rising PVP concentration (from 0.03 to 0.05 g/mL).

If the CdO produced without PVP and the CdO produced with PVP were to be compared, a greater intensity of XRD peaks for the CdO without PVP would be observed. Yet, the benefit of adding PVP can be clearly seen in the eradication of unwanted peaks without the CdO nanoparticles, resulting in improved crystallinity. Essentially, because of the existence of PVP, there is no exchange between ions and PVP chains; this is the process which leads to unwanted peaks of CdO nanoparticles, like the ones demonstrated in Figure 1a. Thus, one of the most vital functions of PVP within the production of CdO nanoparticles is the improvement and stabilization of crystallinity, by lowering or eradicating the extremities of unwanted peaks of cadmium oxide.

#### 3.2.2. Surface Morphology

**Figure 2:**
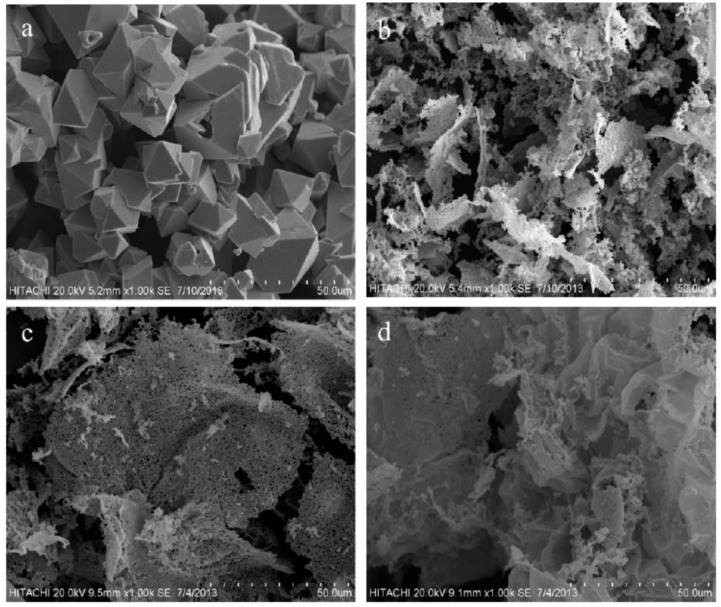
SEM images (50 μm) of CdO nanoparticles at PVP different concentration of (**a**) 0.00; (**b**) 0.03; (**c**) 0.04, and (**d**) 0.05 g/mL.

The surface morphology of CdO nanoparticles has been examined via the use of a scanning electron microscope (SEM), as demonstrated in Figure 2 both with and without PVP. The samples were gathered using a heat based treatment technique. The micrographs were recorded at an electron operating voltage of 20 kV. The production of CdO at 600 °C temperatures without PVP is presented in Figure 2a. It was revealed that the structures were almost completely homogenously dispersed and cubical in shape and oblique crystal form [62]. In Figure 2b–d, the SEM recordings of CdO nanoparticles were gathered in combination with a PVP concentration spanning from 0.03–0.05 g/mL. The findings demonstrate that the morphology was made up of partly nonporous and partly nanosheet shapes [63,64]. Figure 2b,c represents a composite with some nonporous and some nanosheet shapes. The amount of nanosheet forms rose in accordance with a rise in PVP concentration, as demonstrated in Figure 2c,d. The CdO nanosheet forms almost turned into bigger sheet forms in proportional relation to gradual increases in PVP concentration, as displayed in Figure 2d.

#### 3.2.3. TEM Study

**Figure 3:**
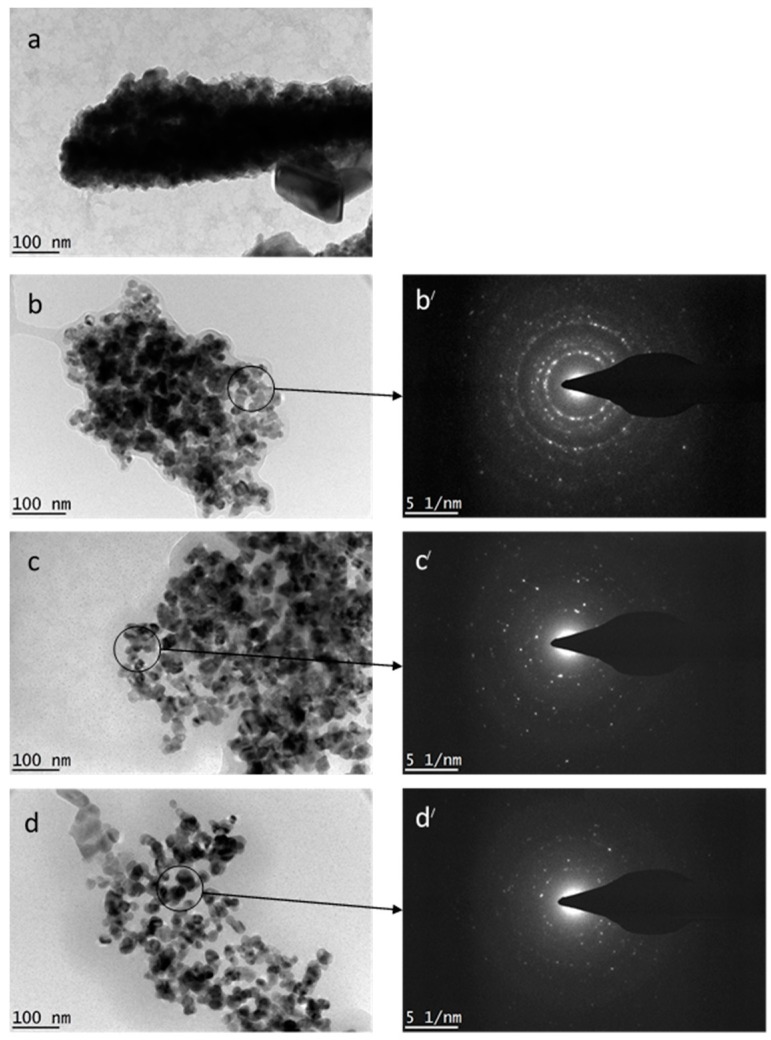
TEM images of CdO nanoparticles and the corresponding SAED at PVP different concentration of (**a**) 0.00, (**b**,**b’**) 0.03, (**c**,**c’**) 0.04, and (**d**,**d’**) 0.05 g/mL.

The cadmium oxide nanoparticles which were readied using a water based liquid (incorporating metal nitrates and a variety of different concentrations of polyvinylpyrrolidone) (*i.e.*, 0, 0.03, 0.04, and 0.05 g/mL) as a capping agent, in order to stabilize the particles and lower the rate of accretion were examined using TEM. Figure 3a demonstrates the images for the CdO sample prepared without PVP, using a heat based treatment technique, at 600 °C. The CdO nanoparticles did form, even without the help of PVP. However, in these conditions, it was observed that the nanoparticles lacked a homogenous dispersion of form and were unevenly accumulated in some areas and not others. In basic terms, the particles were dispersed without balance or an even spread. Thus, it is clear that, without the presence of PVP within the production of nanoparticles, the smaller particles accumulate and eventually turn into larger ones, as a result of elevated surface energy. Plus, the image for some areas recorded melting uniform morphology and a vagueness as to particle size dispersal—this is due to the lack of PVP. The typical particle size at a calcination temperature of 600 °C is tricky to observe via the use of just ImageJ or other imaging software. Figure 3b–d demonstrates the TEM images for particle size and particle size dispersal in CdO samples readied with PVP at concentrations 0.03, 0.04, and 0.05 g/mL and calcined 600 °C. The findings indicated homogenous morphology and even particle size dispersal. The typical particle size at concentrations of PVP spanning from 0.03 to 0.05 mL/g is about 37 ± 3 and 23 ± 3 nm, respectively. Selective area electron diffraction (SAED, Figure 3b,c) were also taken to confirm polycrystalline nanostructure. These results are in line with findings from the XRD measurements calculations. The particles are spherical or elliptical in form, similar to those recorded in previous studies [65,66]. In addition, the findings also suggest that the recorded particle size got smaller, in accordance with a rising amount of PVP concentration. This can be explained by the fact that, as the PVP got stronger, the bigger particles became restricted (or capped) and expansion and accretion was slowed to produce the particles. Also, the restriction of accretion and the decrease of accumulation in samples with PVP lead to cap particles, as long as the PVP concentration is high enough. If contrasted with the concentration of PVP when it was boosted to 0.03 g/mL, it is clear to see that the CdO nanoparticles that formed had a typical size of 37 ± 3 nm. They gradually became more homogenous in form; more so than in conditions without PVP (refer to Figure 3b). Yet, due to the still fairly low concentration of PVP, the nanoparticles accreted anyway—there was not enough PVP to cap them and restrict their accumulation. If the PVP concentration is raised to 0.05 g/mL, the CdO nanoparticles found it difficult to accumulate and were almost homogenous in form, as demonstrated by Figure 3c,d. Nevertheless, in these conditions, the CdO nanoparticles reduced in size from 32 to 23 nm (refer to Table 1). These findings were alike if a PVP concentration of 0.04 and 0.05 g/mL was employed.

The TEM findings of the developed cadmium oxide nanoparticles demonstrate a lower level of accretion within the particles and a much more standardized form when produced with PVP, as a method of capping and limitation throughout the process. The heat based treatment technique also provides the benefit of producing cadmium oxide with an almost homogenous structure and particle size dispersal. The function of PVP is essential for regulating the expansion of the nanoparticles and lowering the rate of accretion.

#### 3.2.4. Phase Composition

**Figure 4:**
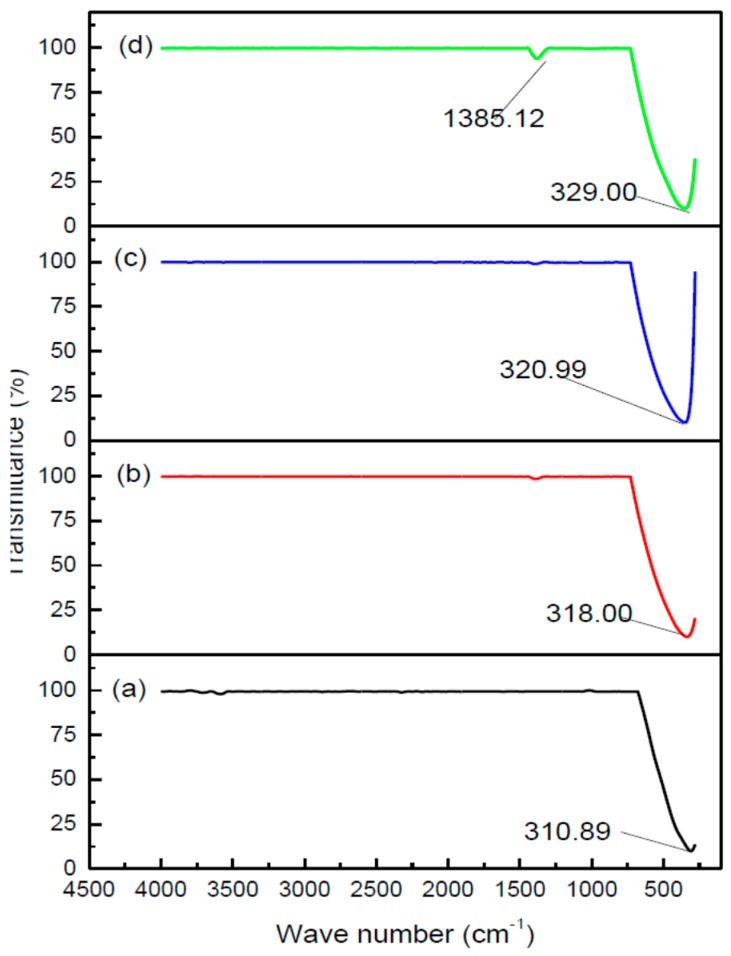
FT-IR spectra of CdO nanoparticles at PVP different concentration of (**a**) 0.00, (**b**) 0.03, (**c**) 0.04, and (**d**) 0.05 g/mL in the range of 280–4500 cm^−1^.

In this CdO nanoparticles investigation, FTIR examinations were used to work out the ideal PVP concentration at which nanoparticles appear, without any organic trace agent present. Examining the exchange between CdO nanoparticles and PVP can also identify this investigation. The spectra results in Figure 4 represent the inorganic and organic components of the samples with a PVP concentration spanning from 0.0–0.05 g/mL over the wave number range of 280–4000 cm^−1^. Figure 4a displays the solo peaks, which can be explained by the presence of metal oxide, in the absence of PVP. Figure 4b,d displays the solo peaks, which can be explained by cadmium oxide nanoparticles with the help of a PVP concentration. The existence of single absorption peak within the CdO spectra values indicates that the CdO were gathered via the use of a heat based treatment technique. The existence of single absorption peaks and the recorded change in the wave number for the CdO nanoparticles spectra values (in the presence of rising PVP) can be explained by the accelerated crystallinity of the CdO nanoparticles, which was achieved via the use of heat based treatment techniques and PVP concentration.

If the concentration of PVP is below 0.05 g/mL, the cadmium oxide nanoparticles which form are as pure as those represented by the values in Figure 4c. Although, small amounts of organic material recorded at 1385.00 cm^−1^ (and attributed to C–H bending vibrations) did appear within PVP concentrations of 0.05 g/mL, as demonstrated in Figure 4d. This indicates that the cadmium oxide nanoparticles were overcome with extreme amounts of the organic components. Thus, for the heat based treatment technique, it is clear to see that the optimum concentration of PVP for the formation of pure cadmium oxide nanoparticles is 0.04 g/mL. This PVP concentration, when brought together with an ideal temperature of 600 °C, offers the circumstances necessary to form pure cadmium oxide nanoparticles with the smallest particle sizes and an improved degree of crystallinity.

#### 3.2.5. UV–Vis Study

The impact of varying concentrations of PVP and 600 °C calcination temperatures on the optical characteristics of CdO nanoparticles have been analyzed and will be outlined in the following section.

**Figure 5:**
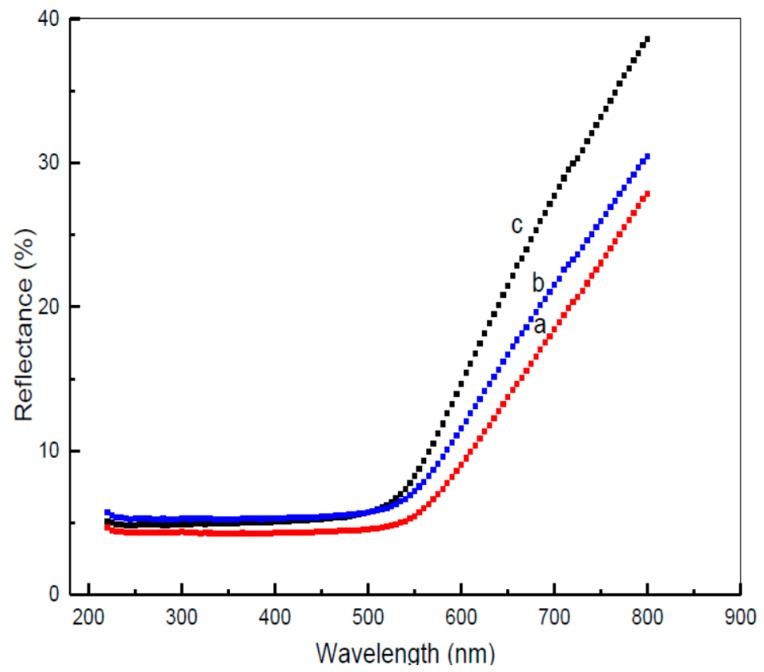
The diffuse reflectance spectra of CdO nanoparticles at PVP different concentration of (**a**) 0.03, (**b**) 0.04, and (**c**) 0.05 g/mL.

The diffuse reflectance spectra values, displayed in Figure 5, were investigated within the range of 200–800 nm at room temperature, for each simple. In the formation of CdO (Cd^2+^ O^2−^), which is ionic compound, 5s^2^ electrons of Cd are donated to O to complete its outer electrons from 2p^4^ to 2p^6^ electrons. In formation of ionic solid CdO semiconductor nanoparticles the semiconductor property of the nanomaterials comes from transition of electrons from valence band (form 4d^10^ of Cd or 2p^6^ of O) to empty conduction band. The samples demonstrate one distinct band within the clearly visible areas, with maxima situated at between 543 and 587 nm that is ascribed Cd^2+^ species present in samples [17]. The rise in extremity of this reflects with the yellow-shift suggests a boost in particle size for the surface cadmium oxide nanoparticles with a lower PVP concentration.

The values in Figure 5 were utilized as a way to determine the absorption coefficient, in line with the Kubelka-Munk (KM) equation [67]. The equation is outlined below:

(2) *F(R∞)* = *α*/*s* = *(1* − *R∞)*/*2R*

where “α” is the absorption coefficient, “*S*” is the scattering coefficient, and *F* (*R*_∞_) is the KM function [68]. For the diffused reflectance spectra values, the KM function can be employed in place of “*α*” for the prediction of the optical absorption edge energy. It was revealed that a plot of *F*(*R*_∞_)*E vs. E* was linear near the edge for direct allowed transition (η = 1/2). The intercept of the line on abscissa (*F*(*R*_∞_)*E* = 0) provided the value of optical absorption edge energy for all the samples.

**Figure 6:**
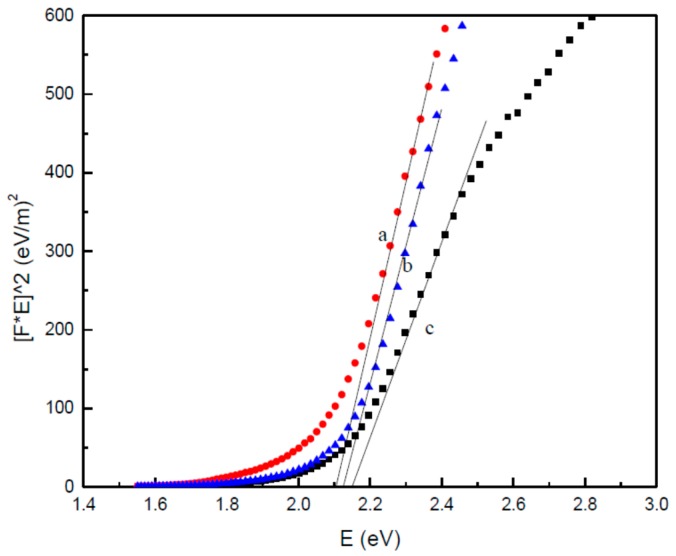
The method of extracting the band gaps of CdO nanoparticles at PVP different concentration of (**a**) 0.03, (**b**) 0.04, and (**c**) 0.05 g/mL.

The optical band gap results for each sample calcined at a 600 °C temperature and varying concentrations of PVP were calculated using the reflectance spectra values and the Kubelka-Munk Equation (3):

(3) (*F*(*R*_∞_). *hv*)^2^ = (*A*(*hv*-*E*_g_))

where *F*(*R*_∞_) is the so-called remission parameter or Kubelka-Munk function, (*hv*) is the incident photon energy, *A* is a constant reliant on the transition likelihood [69] and the diffuse reflectance (*R*_∞_), (*R*_∞_) is the diffuse reflectance which is gathered from *R*_∞_ = *R*_sample_/*R*_standard_ [70]. The values of (*F*(*R*_∞_). *hv*)^2^ *vs.* (*hv*) were displayed as shown in Figure 6. The straight line ranges on the graph have been lengthened to reach the (*hv*) axis [71,72], so as to calculate the ideal band gap values of the CdO nanoparticles at various concentrations of PVP.

It was observed that the optical band gap expanded, alongside rising amounts of PVP, spanning from 2.11 eV at 0.03 g/mL to 2.15 eV at 0.05 g/mL, as demonstrated in Figure 6. The expansion in the energy band gap—in line with rising PVP—can be explained by a reduction in particle size and crystallinity rates, as indicated by XRD values. It is thought that, as the particle size drops, the amount of atoms needed to form a particle also reduces, which then makes the valence and conduction electrons less appealing the ion core of the particles. The eventual result is an expansion within the band gap of the particles.

In contrast, the energy band gaps were revealed to expand alongside a rise in PVP, as demonstrated in Figure 6 (refer to Table 1). The band gap values of the CdO nanoparticles formed at varying concentrations of PVP were observed to increase, as seen in Figure 6. The transition in the values of the band gap was also revealed to have been the result of CdO particle size. The increases in the band gap might also be explained by shifts between the partly concealed valence and conduction bands of the d-shell electrons of Cd^2+^ ions.

The particle size impact on the band gap should not be disregarded. As a result of falling particle sizes, there is a shift in the band structure and within the characteristics of the material. As the size of the nanoparticles lowers, the band gap increases. Therefore, this suggests that at elevated energy settings, the conduction band of of s-electrons and p-electrons are secured, but not in close proximity to one another and at fairly small sizes. At a position closer to the Fermi level—which turns out to be very distant from the middle of the particle—The nuclear potential of the conduction electrons is significant and all shifts involving permitted quantum numbers will demonstrate reduced absorption energy, equivalent to the conduction band energy.

#### 3.2.6. Photoluminescence Study

For this section, the energy level within the structure of a semiconductor of CdO nanoparticles is examined via the use of photoluminescence (PL). The degree of luminescence was evaluated using a spectrometer and the plot peaks across the spectra values reflect a direct calculation of the energy levels within the samples. The samples were prepared with a heat based treatment technique and varying concentrations of PVP.

**Figure 7:**
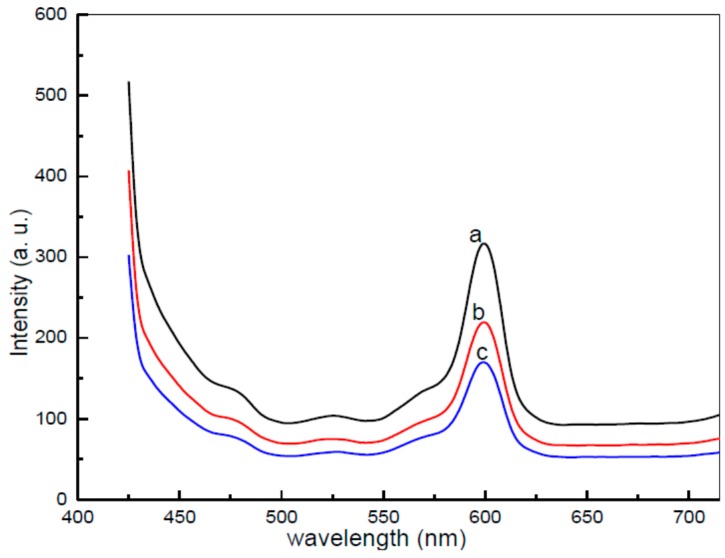
PL spectra of the CdO nanoparticles calcined at 600 °C in different concentration (**a**) for 0.03, (**b**) 0.04, and (**c**) 0.05 g/mL PVP.

Figure 7 represents the photoluminescence spectra values of CdO nanoparticles, as a function of wavelength under excitation wavelength of 425 nm at room temperature. Ordinarily, the average semiconductor of meal oxides supposes a range of prospective emissions, as a result of impurities or crystal defects, such as (1) band-edge recombination; (2) free excitonic transition; (3) exciton band to neutral donor; (4) exciton band to neutral acceptor; (5) donor to acceptor recombination; (6) excitation from interstitial X to conduction band; (7) interstitial M to valance band; (8) X vacancy to the valance band; and (9) excitation from M vacancy to the valance band.

The PL spectra values of CdO nanoparticles created with the help of varying concentrations of PVP at a 600 °C temperature were observed at ambient temperatures under excitation of 425 nm. As demonstrated by Figure 7, the PL spectra values for the CdO nanoparticles prepared with 0.03 g/mL of PVP exhibit a wide ranging emission, spanning from ~470 to 530 nm. Ultimately, this is due to the “composite” impact and the energy states which exist between the valence and conduction bands. These large plot peaks are made up of a couple of smaller sub bands at ~460 and 525 nm [65,73]. The first peak can be explained by the recombination of electron-hole pairs within oxygen and Cd vacancies, respectively [74]. For the second, distinct composites (blue-green emissions observed at 520 nm) were plainly recorded within the PL spectra values of CdO nanoparticles, because of the transition between valence and the conduction bands [66]. The peak within the wavelength span of 600 nm is linked to the deep energy emission levels of CdO. This is a result of intrinsic defects within CdO nanoparticles. If the various concentrations of PVP are contrasted, it is clear to see that gradual increases to PVP lead to gradual reductions in intensity, on top of smaller particle sizes. If the peak with the greatest intensity is contrasted with less intense spectral bands in PVP of less than 0.05 g/mL, it is observed that the CdO particles present a robust cubic structure and only a small number of interior and surface defects.

#### 3.2.7. Electron Spin Resonance

**Figure 8:**
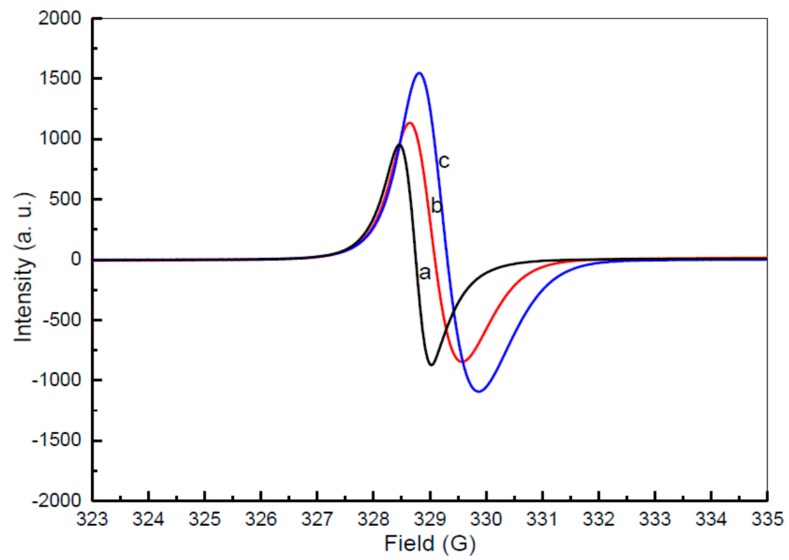
ESR spectra of CdO nanoparticles at different concentration of PVP (**a**) 0.03, (**b**) 0.04, and (**c**) 0.05 g/mL.

The Electron Spin Resonance (ESR) spectrum values represented in Figure 8 are for samples with a PVP spanning from 0.03–0.50 g/mL. A series of wide ranging and regularly occurring indicators were demonstrated by each sample, at a range of different concentrations, as a result of the existence of unpaired conduction electrons of CdO semiconductor nanoparticles. The ESR signal originates either from some sort of paramagnetic defect state within the CdO or from some sort of paramagnetic contamination of the sample. This suggests that the samples were exhibiting paramagnetic characteristics [75]. The resonant magnetic field rose in intensity from 328.764 to 329.294 G, in accordance with a rise in the PVP concentration, from 0.03 to 0.05 g/mL (see Table 2). The values of the 𝑔-factor were reduced from 1.99513 to 1.99235, as the PVP concentration rose from 0.03 to 0.05 g/mL (see Table 2). This suggests that the interior magnetic field reduced due to a rise in PVP concentration, from 0.03 to 0.05 g/mL. This, in turn, indicates that microscopic magnetic exchanges become less abundant decrease as particle size drops. The value of the 𝑔-factor can be calculated using the equation outlined below:

(4) 𝑔 = (*hv*)/(β·𝐻𝑟)

If *h* is Planck’s constant, *v* is the microwave frequency, β is the Bohr magneton (9.274 × 10^−24^ J·T^−1^), and *H*r is the resonant magnetic field. The resonance magnetic field should, predictably, get smaller as the 𝑔-factor gets bigger. However, the value of *v* always remains the same within EPR spectroscopy. Thus, rises in the 𝑔-factor and reductions in the *H*r, in line with a rise in the magnetization values, were observed in earlier investigations of CdO nanoparticles [76].

## 4. Conclusions

This study summarized that the impact and function of PVP within the preparation of CdO nanoparticles (and involving heat treatment techniques) is not just significant, but vital. For a short time, as was outlined during the analysis of XRD results, TEM images, and FTIR spectra results, PVP takes on four essential functions within the production of CdO nanoparticles. These are as follows: (1) regulates the expansion nucleation of nanoparticles dependent on the concentration of PVP; (2) limits the accretion of the nanoparticles; (3) improves the degree of crystallinity of the nanoparticles; and (4) facilitates the development of nanoparticles with a homogenous dispersal of size and form.

The ideal PVP concentration of used was 0.04 g/mL, as this concentration turned out to be the lowest at which the nanoparticles stayed pure. Plus, this was the concentration at which the nanoparticles exhibited smaller particle size and an almost homogenous dispersal of shapes.

This product could be useful for solar cells or sensors because many combinations of CdO at different PVP concentrations would produce different sizes of CdO nanostructures that lead to different band gap values that can absorb multiple wavelengths of solar energy for ideal solar cell application.

## Figures and Tables

**Table 1:** Summary of the structural and optical properties of synthesized CdO nanoparticles at PVP different concentration.

CdO Semiconductor NPs	PVP Concentration (g/mL)	Intensity (%)	*D*_XRD_ (nm)	*D*_TEM_ (nm)	*E*g (eV)
CdO SNPs 1	0.00	9,636	–	–	–
CdO SNPs 2	0.03	4,958	38	37 ± 3	2.11
CdO SNPs 3	0.04	4,804	31	32 ± 2	2.13
CdO SNPs 4	0.05	4,277	22	23 ± 3	2.15

**Table 2:** Magnetic parameters of CdO nanoparticles observed for ESR analysis.

PVP (g/mL)	𝑔-Factor	Hr (Oe)
0.00	–	–
0.03	1.99513	328.764
0.04	1.99287	329.099
0.05	1.99235	329.294
